# “Sometimes I walk and walk, hoping to get some peace.” Dealing with hearing voices and sounds nobody else hears

**DOI:** 10.3402/qhw.v9.23069

**Published:** 2014-03-26

**Authors:** Anne Martha Kalhovde, Ingunn Elstad, Anne-Grethe Talseth

**Affiliations:** 1 Department of General Psychiatry, University Hospital of North Norway, Åsgårdveien, Tromsø, Norway; 2 Department of Health and Care Sciences, Faculty of Health Sciences, University in Tromsø, The Artic University of Norway, Tromsø, Norway; 3 Jaeren District Psychiatric Center, Bryne, Norway

**Keywords:** Hermeneutics, lived experiences, auditory hallucinations, coping, mental health nursing

## Abstract

Our objective in this article is to add to the understanding of how people with mental illness experience dealing with hearing troublesome voices and sounds in everyday life. Fourteen people contributed through in-depth interviews and we analysed these using a hermeneutic phenomenological approach. We found that the participants (a) tried to block out the voices and sounds, (b) navigated the health care services, and (c) struggled to come to terms with limitations. Our overall understanding of how the participants dealt with hearing voices is that they sought to be independent and lead ordinary lives despite being troubled by voices. The participants fought desperately to find relief and avoid being overcome by the voices and sounds in intense phases. In less intense phases, they developed ways of getting along with daily life in spite of these experiences. We reflect on the implications of these findings and emphasize the need for care providers to attempt to understand and engage in collaborative explorations with service users in search of the most helpful ways of dealing with hearing troublesome voices and sounds in everyday life.

Persistently hearing troubling voices and sounds that others do not hear can seriously disrupt the everyday lives of those who have these experiences (and their families). The phenomenon is associated with educational difficulties, diminished ability to work, and suicidal behavior (Harkavy-Friedman et al., 2003; Kalhovde, Elstad & Talseth, 2013). Experiences of hearing voices, commonly known as auditory (verbal) hallucinations, are regarded as a hallmark symptom of serious mental illness in Western cultures. These experiences are predominant among people diagnosed with a schizophrenia spectrum disorder and other psychotic disorders (Waters et al., 2012).

Researchers have been increasingly interested in how people cope with symptoms and distress related to chronic illness and in developing approaches aimed at enhancing the coping skills of service users (e.g., cognitive behavioral therapy). Approaches focused on improving coping with voices have shown promising but not convincing results (Farhall, Greenwood, & Jackson, 2007). Despite long-term pharmaceutical and psychosocial interventions, many remain seriously troubled by hearing voices (Mueser & McGurk, 2004).

The first author of the present article (AMK) was challenged by voice hearers’ stories of lonesome struggles to manage everyday life with voices and sounds in an earlier study (Kalhovde, 2005) and began to wonder how care providers can lessen the burden of their struggle. Consumer movements (e.g., the international network InterVoice) argue that health carers’ understandings and goals have not been attuned to voice hearers’ understandings, goals, and preferences in dealing with the voices (Escher & Romme, 2012; InterVoice, 2012). Lakeman (2001) suggests that nurses might unwittingly hinder service users’ attempts to cope and might undermine their sense of self-efficacy if their interventions are not based on sensitivity to the person's distress, understandings of their voice experiences, and the person's ways of dealing with them. Fenekou and Georgaca (2010) also argued that health carers should be attentive to and engage in improving the explanatory frameworks and coping strategies developed by voice hearers with long-term experience. Few researchers have focused explicitly on how voice hearers experience dealing with voices under shifting circumstances and in different phases of their everyday lives (cf. Farhall et al., 2007). Romme and Escher (1989) suggested that voice hearers’ coping strategies could be grouped into (a) a startling phase characterized by escape from fear and anxiety, (b) an organizational phase seeking meaning and understanding in the voices, and (c) a stabilization phase in which more permanent ways of dealing with voices are acquired.

In a previous publication (Kalhovde et al., 2013.), we explored how people with mental illness experienced hearing voices in everyday life. The participants’ daily lives were recurrently dominated by intrusive and opposing voices. The participants were varyingly convinced that they heard someone else or themselves and they were terrified that this meant they were losing their minds. The tones and contents of the voices echoed and amplified past, present, and future experiences and worries, and came forth as the intentions of others intrusively resounding in the participants. In the present article, we pose the following research question: how do people with mental illness experience dealing with hearing voices and sounds in everyday life?

## Methods

People with lived experience of hearing voices and sounds and who had been diagnosed with a psychotic disorder participated in in-depth interviews conducted by the first author. Inspired by Gadamer's philosophy (2004), we analysed and interpreted the transcribed interviews with a hermeneutical phenomenological approach. Gadamer distinguished between conversations, in which our aim is to know the other person and his horizon (e.g., a therapeutic conversation), and true conversations, in which we aim to recognize the other person's claim to truth e.g., in-depth interviews about a subject matter (Gadamer 2004, p. 303). These perspectives are particularly relevant for research (and clinical practice) in relation to the experiences of people who are categorized as being mentally ill. Gadamer (2004) emphasized that the hermeneutic circle is not a methodological circle but describes the ontological structure of understanding. This structure is determined through our anticipatory movement of preunderstanding (Gadamer, 2004, pp. 293–294). Our only chance of reaching beyond the confines of our own assumptions lies in exposing ourselves to opposing views (Gonzalez, 2006). This exposure is a fundamental part of the hermeneutic experience and is essentially negative, since we are continually dismissing false generalizations. Gadamer argued that this negativity is fruitful because we then acquire more comprehensive knowledge (Gadamer, 2004, pp. 347–348; Gonzalez, 2006). Thus, our hermeneutic understanding of a text is dialogically structured through questioning and answering. The defining property of questioning is to open up possibilities and to keep them open (Gadamer, 2004, p. 298).

### Participants

Health care providers recruited the participants through community-based (five) and out-patient (seven) mental health services in Norway from 2008 to 2010. They conveyed oral and written information about the research project to adult service users who met the following criteria: (a) were hearing or had heard voices and sounds that they alone experienced for at least a year and (b) had been diagnosed with a psychotic disorder. Those interested in participating in the study contacted AMK directly. Two people volunteered after reading about the project in the media; one of these was not receiving treatment at the time and was therefore included after consultation with the ethics committee. The other person conferred with his therapist who provided him with written information and a consent form for the study. AMK was acquainted with three of the participants prior to participation; however, their participation in the study had not been discussed before they volunteered.

Fourteen people (eight women and six men; age range: 19–57; median age: 39) who had been hearing voices and sounds for 2–39 years (age range of first voice experience: 8–32; median age: 16) were included. The participants reported having diagnoses in the schizophrenia spectrum (nine) or combinations of other different diagnoses, such as personality disorder, posttraumatic stress disorder (PTSD), and depressive psychosis (three). One participant was initially diagnosed with schizophrenia and was being reassessed for PTSD at the time of the study. One participant was unable to disclose a diagnosis.

Care was taken to inform the participants of possible reactions the interviews might trigger (e.g., strong emotions and increased voice hearing) and AMK offered to assist the participants in contacting the recruiting clinician if they desired. None of the participants requested assistance or withdrew from the project. We carefully modified all identifying characteristics to avoid identification of the participants while maintaining the information related to the objectives of the study. The Regional Committee for Medical and Health Research Ethics in Northern-Norway approved the research project.

### Interviews

The participants were asked to relate what it was like for them to live with hearing voices or sounds. AMK posed follow-up questions to clarify particulars and encourage the participants to elaborate on matters that seemed relevant, and aimed at reflective statements to ensure mutual understanding. Questions from an interview guide were also used as prompts, if required. AMK was dedicated to learning from all the participants and did not seek to confirm presuppositions or emerging theories (cf. Binding & Tapp, 2008 on genuine dialogue; Gadamer, 2004 on true conversation). Our intent was that each interview should contribute to our hermeneutic learning process. The interviews were digitally recorded and transcribed verbatim, with one exception. In this interview, notes were made throughout and immediately after the interview because the interviewee objected to having the first interview recorded.

AMK and the participants established the location, number, and timing of the interviews according to the participants’ preferences, within a limit of three for each participant. Most participants (nine) took part in two interviews; three participated in one; and two participated in three. The total interview time for each participant was approximately 1.5–2 hours. Most follow-ups took place within a month (11), whereas three took place within 6 months. The follow-up interviews provided the participants and AMK with the opportunity to reflect on and elaborate on relevant matters with less risk of exhausting the participants. Two interviews took place at the workplace of AMK, and the rest (12) took place in the interviewees’ homes. One follow-up was conducted by telephone as requested by the participant.

### Text analysis

We analysed the interview texts inspired by the four cyclical steps outlined by Fleming, Giadys, and Robb (2003). These steps concur with the hermeneutical circling in Gadamer's hermeneutics (2004) as previously described. First, an overall understanding of each text unit in relation to the research question was formed in writing by the first author (cf. Fleming et al., 2003). The text units comprised transcripts of the interviews and follow-ups as well as notes taken subsequently concerning each participant. Once the recording was transcribed, we began to analyse each text unit. AMK listened to and reflected on the recordings, and all the authors read and reread the texts. Our further questioning and in-depth interpretations of the texts were based on this preliminary understanding. The second and third authors are nurses experienced in the fields of qualitative inquiry, philosophy, and the education of nurses and mental health care providers.

Second, we marked all meaning units (paragraphs, sentences, or parts of phrases) and reflected on them together to reveal similarities and nuances of how the participant in question dealt with the voices and sounds (cf. Fleming et al., 2003). Written interpretations facilitated the formation of themes and subthemes in the ongoing dialog (cf. Binding & Tapp, 2008). Occasionally when we met, we developed themes together using the blackboard. During the analysis, we continually discussed each other's presuppositions and interpretations.

Third, we compared the themes and subthemes with the written overall understanding of each text unit and adjusted them (Fleming et al., 2003). Then we developed and put into writing our comprehensive understanding comprising the commonalities and distinctions of all participants. We read the themes and subthemes related to all participants successively, and assembled and compared them with each other and the comprehensive summary, before revising the summary. Finally, we included quotes that highlighted our understanding of the phenomena in question (cf. Fleming et al., 2003). In addition, we reflected on the results in relation to relevant literature. We completed the analysis when we had acquired a shared understanding, namely when the participants’ and our own perspectives had become integrated, and our understanding of the entire text corresponded with our understanding of its parts. The computer software NVivo 8 (QSR International, 2008, Victoria, Australia) was used in the early phases of the analysis and to organize the notes AMK made during the study.

## Results

The participants were in what we understood to be different phases of highly personal trajectories that included long-term experience with mental health services. For some participants, the initial, intermediate, and final phases of dealing with the voices occurred in a linear pattern and for other participants the phases followed cyclical patterns, in which the participants’ attention was repeatedly and erratically claimed by upsetting voices and sounds. Consequently, the participants were at different stages in their reflections and in different stages of relating to hearing voices and sounds at the time of the interviews. Many participants also heard benevolent voices, but these voices did not demand their attention and therefore the participants had no need to deal with them. We present the results concerning how the participants dealt with hearing voices and sounds in everyday life through the following themes and subthemes, structured in the above mentioned phases.

### Trying to block out the voices and sounds

The following subthemes illuminate the different facets encompassed by the participants’ initial attempts to block out the voices and sounds: (a) keeping busy and carrying on with daily life as usual, (b) avoiding talking about hearing voices and sounds, and (c) resorting to desperate measures to achieve relief.

#### Keeping busy and trying to carry on as usual

Initially, most participants blocked out the voices and sounds by constantly being in activity and allowing little time for rest or breaks, in which their attention could be claimed by the voices and sounds. Some participants kept themselves preoccupied with sorting out other adversities they were encountering. Most of the participants managed to maintain their daily routines for months and some participants for years (e.g., by engaging in school, work, family, volunteer work). One participant explained:

‘cause it was like when I had something to do all the time and concentrated on something else, then I sort of didn't hear it . … All I did was, like, work and sleep and go to school and then party at weekends . … I didn't want to be left alone,’ cause that's when I was, like, bothered.

Some participants were indifferent to the voice messages and could thus carry on as usual with little effort.

#### Avoiding talking about hearing voices and sounds

Most participants did not speak about their experiences of voices and sounds for years once they had established that they alone heard them. Thus, these participants attempted to maintain everyday life as usual and carefully avoided disclosing information that could lead others to doubt their sanity. One participant preferred her family to believe that she was merely depressed. Another participant let others know about her addiction to drugs but not the voices she constantly heard. “I didn't tell anyone about the voices then, because it was completely like: Okay! I was a pill junkie, but being crazy, no, I didn't want that.” Some participants avoided disclosing sensitive matters in general; most participants did not reveal their experiences with hearing voices and sounds in particular, even while receiving mental health care. Some participants had experienced that their trust had been breached by health care providers, who had informed their parents. Several participants also avoided talking about their voice experiences to avoid disclosing traumatic or shameful experiences (e.g., sexual and physical abuse, parental neglect). Some of these feared that the threats expressed by their violators and the voices would be carried out if they revealed what they heard. Several participants evaded conversations about hearing voices because this could increase troublesome voice hearing.

Most participants initially withheld information about the voices because they were adolescents at the time and wanted to manage on their own or avoid meddling by parents and health care providers. The participants who were parents themselves concealed their voice experiences because they feared they could lose custody of their children.

Then, I contacted the child protection services and received home visits from a psychiatric nurse . … We talked together once a month I think, but I didn't dare to say that I had voices, then, (…) cause I was afraid they would take my baby from me.

Most of the participants had not yet spoken about hearing voices with family members, friends, and even trusted fellow patients and friends.

#### Resorting to desperate measures to achieve relief

Sooner or later, the participants became so exhausted and ill, or the voice experiences became so agonizing, that the participants were unable to reason and divert their attention from the voices and sounds. The participants’ main objective then became to achieve peace. In search of relief, many took to desperate measures such as self-harm or attempted suicide. “All my attention went to that [hearing voices] and it got so difficult that it became too much (…) then I tried to kill myself.”

One participant harmed himself a couple of times; he had heard that it was common to do so, but did not find it helpful. “I'd heard that many people … harmed themselves, so I tried it just a couple of times. But it didn't do anything for me, so I just quit.” In periods of intensely troublesome voice hearing, a few participants were convinced that other people could hear the intimidating messages they heard. Consequently, they withdrew from family and friends, school, or work, or strictly limited the time they spent with others. One participant began to attack other people at one point in time; he was persuaded by the voices that someone was out to get him and he felt he had to be prepared for an attack.

### Navigating health care services

The next phase involved the following aspects of the participants’ involvement with health care: (a) talking about hearing voices and sounds in due time, and (b) negotiating the wanted and unwanted effects of medication.

#### Talking about hearing voices and sounds in due time

Many participants mentioned to others that they heard voices and sounds before they realized that only they heard them and what that meant. One participant heard her mother explain to health care providers that the participant heard voices and therefore should receive treatment; only then did she realize the significance of what she heard:

When we had that introduction talk my mother said, ‘Yes, she says she hears voices. She says she hears things’ and that's when it first dawned upon me: Wow! What's this? I became aware of the fact that I heard voices.

This participant realized that these were experiences commonly related to mental illness. The participants carefully considered their experiences of voices and sounds and eventually decided to share them with care providers whom they trusted. One participant described how, when he met a care provider who listened to what he had to say, he could cry and the knot inside him was loosened, he was able to sleep and the voices lost their power shortly after. He said,

Sensations from the outside finally came through. … [I] managed to get kind of a lot of positive impressions … there had been so much negative [experiences].

Another participant chose to confide in a care provider who surprised her by inquiring about hearing voices; she had never been asked such questions despite receiving mental health services for many years. This participant found it a relief to be able to talk about the voices and be treated as a person that was accountable despite the fact that she heard voices, “God how wonderful, I thought, finally someone I can trust, sort of, and could tell everything to, and I had controversies and discussions [with].” Some participants eventually also involved certain family members in their experiences of hearing voices. When the voices reoccurred, one participant conferred with a trusted sibling, before she decided to involve a health care provider about what she should do. “First I told my sister and then I told my psychologist and … I asked for sick leave … it [being on sick leave] was good. … I regained my energy.”

Some participants sought health care services for problems they had besides the voice hearing (e.g., eating disorders, intrusive memories from abuse, and addiction to drugs). Nonetheless, nearly all participants were subjected to involuntary hospital admissions as a result of the desperate actions they had taken to gain relief from hearing voices, and because they were overcome by psychosis and rendered unable to take care of themselves. Many participants were disappointed with the health care services they had encountered. Several participants felt obliged to accept the explanations of health care providers, which did not resonate with their own experiences and dismissed their understandings. One participant pointed out that there was little information available on how to deal with voices and was convinced that this was connected to prejudices against the mentally ill. Talking about hearing voices to health care providers led to trials with neuroleptics for all of the participants and taking this medication then became the main issue.

#### Negotiating the wanted and unwanted effects of medication

The participants found that taking neuroleptic medication not only mostly muted the voices and sounds but also led to other highly troublesome plights. Most participants’ physical appearance changed (e.g., gaining weight or developing involuntary movements). One participant explained:

It [neuroleptic medicine] actually worked pretty good, it [the voices] sort of became more like background noise … but I put on ten kilos in one month and I was sixteen and then I refused to take it.

Several participants felt that the neuroleptic medication altered who they were and impaired their ability to manage everyday life and deal with the voices and sounds. The medicine diminished their ability to think, talk, and feel, as one participant explained:

I didn't feel like myself … it [neuroleptica] changed the way I talked, so I talked slower, and didn't remember words. … I was depressed the whole time. It wasn't like it made me happy. Now it shifts more, now that I've quit [using neuroleptics] and I think that's much better, because now I feel more.

Another participant not only became indifferent to the voices but also generally lost interest in life while using a certain brand of neuroleptics. She said, “I became very indifferent to everything on that medicine. It [brand name] should be illegal.” All of the participants had tried out a number of different brands and types of medication. One participant said, “So then I became a guinea pig on meds again … tried most all there is of antipsychotics and antidepressants.” Many participants took neuroleptics because they felt they had no choice. One participant said, “They [health professionals] just stuffed in loads of meds and it was quite clear that I could, I just had to take them or else, of course, it would be forced treatment.”

Most participants who were resigned to long-term use of neuroleptics reduced the prescribed doses. One participant had short medication-free intervals to enable himself to be more reflective and emotional. Thus, he could interpret the voices and the issues they addressed. When challenging circumstances demanded more of him, this participant increased the dosage to avoid becoming psychotic; sometimes he succeeded, at other times he became psychotic and unable to take care of himself. He said:

If I don't manage to balance … then I sometimes get confused and stuff like that. … I would rather have some psychotic symptoms and have an emotional life, than be emotionally dead and not have any symptoms … Then you'll never get anywhere, if I just put a lid on the whole illness with medication.

Several participants realized that sleeping pills were the only type of medication that was moderately helpful, since acquiring enough sleep was the best “medicine” for troubling voices. Others emphasized that antidepressants and anxiolytics were the most helpful. One participant explained that when the voices were harsh, the only thing that helped was to take anxiolytics and go to bed for as long as it took for them to diminish.

### Struggling to come to terms with limitations

The following two subthemes illuminate facets of the final phases of how the participants’ dealt with hearing voices and sounds: (a) learning to live with the voices and sounds by recognizing patterns, (b) approaching acceptance and identifying possibilities, and (c) making sense of hearing voices and sounds.

#### Learning to live with hearing voices and sounds by recognizing patterns

Most participants eventually discerned patterns of how and when the voices and sounds emerged. The participants recognized which voice messages they could resist and which ones they were forced to resign to, depending on how fit or exhausted they were. When the voices were moderately troublesome, one participant merely turned on the radio, engaged in routine activities, such as tidying the kitchen, and became oblivious of the voices. When the voices intensified, several participants resolved to walk. One of them explained:

I try to do what I was meant to do [recommendations from care provider] but sometimes I just give it all up, because everything is, just gets muddled for me, and sometimes I hurry out and walk and walk … hoping to get some peace.

Several of the participants kept diaries in which they expressed their emotions in relation to hearing voices and sorted out matters of importance. One participant expressed her anger toward the voices. Another participant noted the recommendations she received from health care providers. One participant considered writing a book based on his own diary.

The participants found ways of carrying out everyday activities by negotiating how strongly they resisted the commanding voices. One participant took a cold shower in response to the voices that demanded she throw herself in the sea on a cold winter's day. Another participant feigned cutting herself when voices persistently insisted that she hurt herself. A health care provider encouraged one participant to talk back to the voices, but the participant was unable to do so. When attempting to fall asleep while being pestered by voices, she instead contradicted the voices through imagery writing.

She [health care provider] said I should talk to the voices and dismiss them, but that's difficult for me to [do], what I can do in the evenings, this may sound funny, but when the voices dominate my head, I point my finger at the bedclothes and write to them … and answer them that way.

One participant removed the television from his apartment and avoided most radio channels in order not to hear his thoughts and feelings being broadcasted. He had found one channel where he was certain that the hosts would not comment on him, as they had a tone that was encouraging and made him feel good. Another participant arranged for a friend to accompany her on shopping trips to ensure that she withstood the voices demanding that she steal or buy clothes she did not need. This helped the participant even though she had not informed her friend about the voices. Many participants reflected that alcohol and drugs gave temporary relief but ultimately caused the voices to intensify. One participant said, “I notice that it's as if the voices calm down when I eh, drink wine or beer and so on, don't know why, I've given it some thought … but then, afterwards: Oh no!”

Most participants had developed ways of relaxing while enduring periods of troublesome voice hearing by listening to or meditating to music they appreciated. The voices and sounds often became less intense and the participants could divert their attention to this source of enjoyment. Several participants noticed that enjoying themselves or feeling good helped to lessen the burden of hearing voices and sounds. The effect of enjoyment was however not without limits. Several participants balanced how much or how long they enjoyed themselves before the voices became too intense. One participant said,

Now I can manage to watch TV, like those comedy series that last half an hour and if I see all of it, sometimes I do manage to laugh … but I cannot watch movies and long programs, that doesn't work.

Several participants also noticed that when life in general was going well, the voices faded or disappeared. One participant did not hear voices while she was pregnant. She reflected that she might have been so preoccupied with becoming a mother that she took no notice of the voices. Another participant said that taking part in routine activities (e.g., going to the gym or community mental health activity center) gave her life meaning. Leading a meaningful life enabled her to resist the voices telling her to do away with herself.

#### Approaching acceptance and identifying possibilities

A significant change to how many participants dealt with the voices occurred when they decided that if they could not be rid of them, they would no longer let these experiences dominate their lives. One participant angrily decided that enough was enough; she joined a hearing voices group and continued to attend these meetings despite intense voice hearing after each meeting. Another participant decided to stop cutting herself in obedience to the commanding voices she heard because she did not want more scars on her body. Most participants found that being firm rather than hostile toward the voices was more effective. One participant said, “When it [the voices] becomes too much I just say ‘so what’ out loud and that's that.” One participant told the voices he heard to go on vacation. Although these “vacations” seldom lasted more than a couple of days, he was able to enjoy the breaks he achieved.

Most participants wrestled to adjust their aspirations and had not abandoned hope for a brighter future that involved, for example, being employed and having a family and a home of their own. Some participants managed to work part-time or have sheltered jobs despite being troubled by voices because they could take a break and withdraw from others when needed; others found such working conditions were hard to come by and found alternatives. One participant explained that even though he believed that he would never be well again, he would make the most of his life and work hard to get better. He composed music and published it on the Internet hoping to be recognized for his work and live a fairly normal life in the near future. One participant had given up attempting to finish school and hanging on to a job; instead, she chose to be involved in a meeting place for people with mental illness, which she now defined as her workplace. She preferred an environment in which she could be sure that co-workers would not harass her or reject her because she heard voices. Another participant resolved to assist an ill family member, hunting and fishing at a pace he was comfortable with.

Some participants found it liberating to joke about the voices and their own reactions to them. Joking about hearing voices put family, friends, and health care providers more at ease and facilitated relating to them. One participant said, “I usually don't talk about the voices with family. … A joke, like fooling around with it, I can do that, though.” Another participant made fun of a dominating and troublesome voice she heard by giving it all sorts of humiliating names; thus, she acquired a sense of power over the voice and the person the voice resembled. Several participants had attended groups for voice hearers. They found it helpful and reassuring to share their experiences with peers. One participant recounted: “It's like … good to talk about it [hearing voices] and we, like, [have an] agreement that it's a hassle sometimes … it's good to meet others who've experienced those kinds of things too.” Some participants were angry and unable or unwilling to accept the limitations forced upon them by persistently hearing voices.

#### Making sense of hearing voices and sounds

Most of the participants spent a considerable amount of time pondering over the content of the voices and sounds, either because the voices were perceived as helpful or because the participants were struck by the distressing messages. In an attempt to silence the voices which addressed existential questions concerning whether he would be condemned to hell or not in afterlife, one participant sought answers in philosophical books instead of the Bible. A few participants believed that hearing voices was a form of contact or communication with other people who were deceased or absent; by listening to the voice messages these participants could play an important part in society. Several considered the perspectives of health care providers before they found their own stance. One participant did not believe that the voices were related to illness as explained by the health care providers but rather chose to believe that the voice experiences merely indicated that she was different,

They [health care providers] tell me to block it [the voices] out … they think I'm ill … there's a little dispute about that … I believe I'm well … people are different.

Instead of diverting her attention from the voices and seeing the very presence of the voices as signs of her continually being ill, she sometimes listened to them and allowed the voices to fill the painful emptiness she felt in her life. Several participants found that they could gain relief by dealing with the issues the voices addressed. One participant rephrased hostile voice messages and spoke to himself in a softer tone about the matters they addressed, “So what's best for me to do, of course, is to pay a little attention to them [the voices] and interpret them my way. … I don't have to agree 100% with them.”

Another participant reflected that talking about and dealing with the trauma related to the sexual abuse she had endured was somewhat helpful in relation to hearing voices. When she finally decided to disclose and address her experiences of hearing voices in light of her traumatic experiences they actually diminished. Not all the participants had acquired a comprehensive understanding of why they heard voices. One had accepted that she could not understand why she heard voices and what they meant. A few participants believed that the voices were nonsensical symptoms of schizophrenia and the most efficient way of dealing with them was to find the right medication and dosage and ignore them.

## Overall understanding and reflections

Our overall understanding of how the participants dealt with hearing voices is that they sought to be independent and lead ordinary lives despite being troubled by voices. The participants developed ways of dealing with hearing voices and sounds through highly personal trajectories. They fought desperately to avoid being overcome by the voices and sounds in intense phases. In less intense phases, they developed ways of getting along with their everyday lives in spite of these experiences. Some participants eventually accepted that they were recurrently disabled by hearing voices; others struggled relentlessly to be rid them.

We note that the participants were able to avoid focusing on the voices and sounds when they had something meaningful and purposeful to be engaged in (e.g., work, or activities defined as work). These findings corroborate results reported by Delespaul, DeVries, and Van Os (2002, p. 102) suggesting that work was “the most powerful strategy” in reducing the intensity of hearing voices. The participants’ efforts to cling to work or school as a way to avoid addressing the challenges the troublesome voices represented could also be understood in light of the benefits of employment. Although often highly stressful, work is a significant source of identity, a way of structuring time, a resource in coping with hardship, a source of pride, or a worthy adversary (Benner, 1994).

The participants closely considered the benefits and drawbacks of employment and contemplated engaging in sheltered work or volunteer work instead of a competitive job. For many work was only an option if they could work at their own pace and take breaks or withdraw from others when too troubled by voices. These results are corroborated by Honey (2004) who reported that only when the benefits of employment outweighed the drawbacks did the persons with mental illness choose to obtain or maintain employment or prefer volunteer work to a competitive job. Working was not only a way of avoiding the voices but also a motivator in recovering from psychotic crises. These findings coincide with those of Gunnmo and Bergman (2011). We also note that the participants not only described listening to music as an efficient means of distraction, but it also provided them with the opportunity to relax and enjoy themselves and was even a source of acknowledgement. People's experiences of the multifaceted role music might play in recovering from mental illness have recently been described by Solli, Rolvsjord, and Borg (2013). These authors argued that music therapy should be a standard part of mental health services for people with severe mental illness because of the multitude of benefits it can provide.

The desperate measures the participants resolved to when intensely troubled by voices often led to involuntary psychiatric treatment. Incomprehensible behavior is defined as a symptom of psychoses in diagnostic manuals (cf. American Psychiatric Association, 2000) in terms of “disorganized behavior.” We suggest that the participants’ actions also should be understood in light of how desperate they were for relief (cf., Kalhovde et al., 2013). The participants’ desperate actions might also be understood as lack of experience with alternative strategies and lack of trust in any support they anticipated from health services, family, and friends.

Most participants were reluctant to seek health care and were also reluctant to disclose their voice experiences long after their first contact with mental health care services. When people with serious mental illness avoid seeking mental health care, this conduct is often understood as lack of insight into the origin of their troubles (e.g., voices are symptoms of psychosis; cf. American Psychiatric Association, 2000). Most participants in our study did however realize, once they discovered that they alone heard the voices, that these were experiences associated with serious mental illness. Their reasons for not disclosing their experiences were various, such as to avoid revealing abuse, not to risk intensified voice hearing and retaliation from the offenders, or to avoid revealing their most sore and sensitive thoughts and feelings. Roe, Hasson-Ohayon, Kravetz, Yanos, and Lysaker (2008) showed that the illness narratives of people with a schizophrenia diagnosis who appeared to be unaware of having an illness, or who actively rejected their diagnostic label, revealed that they in fact had substantial insight into their illness. Abundant research has shown that the fear of being misunderstood or shamed plays an important role in keeping young persons with serious mental illness from seeking care (Rusch, Angermeyer, & Corrigan, 2005). The participants’ resistance to involving others in their struggle with hearing voices can also be understood as an effort to obtain or maintain independency.

The participants were active in their attempts to relate to health care providers and to find someone they trusted. When they succeeded, it meant they had someone with whom they could share the burden of their experiences and develop new perspectives on hearing voices and ways of dealing with them. These findings agree with Ådnøy Eriksen, Arman, Davidson, Sundfor, and Karlsson (2013), who described three levels of connectedness: (a) being detached, (b) being cautious, and (c) being open and trusting, where the last level was understood to enhance recovery (pp. 3–4). Researchers have highlighted the importance of health care providers establishing trusting relationships (Hewitt & Coffey, 2005; Lorem & Hem, 2012) and asking about experiences that patients seldom volunteer (Bebbington et al., 2011). The need for enhancing trust in mental health care services in general has also been underscored (Rickwood et al., 2007; Rusch et al., 2005). The active part that service users play in this respect has only recently been acknowledged. Topor and Girolamo (2010) have, for instance, revealed that service users with serious mental illnesses were constantly making quality judgements of carers they came in contact with and that they were careful about whom they talked to and what they said.

Based on our results, we suggest that the participants’ dissatisfaction and disengagement regarding health care might be associated with the three aspects described by Kreyenbuhl, Nossel, and Dixon (2009). These authors pointed out that disengagement from treatment reflected the service users’ perceptions that treatment was not necessary, was not meeting their needs, or was not being provided in a collaborative manner. Our results demonstrate that the participants’ efforts to balance the wanted and unwanted effects of using neuroleptic medication rested on complex decisions which they mainly made on their own. Deegan (2003) illuminated how the focus on compliance and non-compliance has failed to capture the dilemmas the participants are struggling with regarding decisions on treatment, especially related to the disabling effects of medication. She has also shown that deciding to take medication is a dynamic journey and not a one-time event. Deegan (2007) contended that health carers should provide ongoing support in decisional conflicts. Roe, Goldblatt, Baloush-Klienman, Swarbrick, and Davidson (2009) shed light on the dynamic interpersonal context of the process of choosing to stop taking medication. There is an emerging awareness that the effects of neuroleptics are highly personal and that health care providers need to inform and involve patients in decisions regarding the need for trial and error (Tandon et al., 2008) when attempting to find the best pharmaceutical treatment (Lorem, Frafjord, Steffensen, & Wang, 2013). These researchers all point to shared decision-making as a pathway to enhancing collaboration and assisting the service user in making informed decisions.

Several participants emphasized the importance of receiving peer support. Deegan (2003) contended that participating in peer groups in addition to treatment enhances the empowerment of people in their recovery processes. Settings such as self-help groups can also enable voice hearers to make sense of their experiences and develop their own understanding without being limited to the medical model (Blackman, 2000; Thomas, Bracken, & Leudar, 2004). Voice hearers have argued that the medical model was disempowering because it rendered them with little hope of recovery and limited possibilities of contributing to this process (Escher & Romme, 2012). These authors also emphasize the role peer groups can play in reducing isolation.

Many participants in our study had begun to reassess their situation, found ways of engaging in or maintaining activities they enjoyed or found meaningful, and adjusted their future aspirations despite persistent suffering. Other participants were bitter and angry at the turn their lives had taken and the loss of opportunities and were engaged in resisting troublesome and often intense voice hearing on a daily basis. These findings correspond with the results from a former study (Kalhovde, 2005) in which a participant said, “Being able to read a book while hearing voices without being troubled by it, that's living with [voices]. If you have to get rid of the voices first, before you read that book, then it's, like, living against [the voices]” (p. 117). These findings also agree with Ironside et al. (2003) who found that “letting go” was a fundamental aspect of living with chronic illness. This did not involve giving up, but knowing when to let go of control and when to control. These authors found that the participants’ accounts of “coming to terms with” and “accepting” could be described as “living into a future of new possibilities” or “being attuned to new possibilities” instead of resigning to the functional limits imposed on them (p. 179). Researchers have recently shown increasing interest in the process of acceptance as an essential aspect of living with chronic or recurring conditions and emphasize that health carers should address and facilitate the process of acceptance in relation to (e.g., chronic pain) (Viane et al., 2003). Nilsen and Elstad (2009) showed that when persons with chronic pain had the opportunity to convey storylines from their trajectory, it helped them to look forward and gave room for hope. An increasing number of researchers argue that health carers should assist voice hearers in exploring and attempting to understand what the voice experiences mean in the context of their lives and thus enhance coping and acceptance (Beavan & Read, 2010; Kalhovde et al., 2013). Romme, Escher, Honig, and Noorthoorn (1992) have produced a questionnaire to assist care providers and voice hearers in developing detailed narratives which can enhance comprehensive and mutual understanding (cf. Romme, Escher, Dillon, Corstens, & Morris, 2009). Several participants in our study found writing to be helpful in dealing with hearing voices. Creative and expressive writing has been shown to enhance reflection and the writers’ understanding of thoughts and feelings (Furnes & Dysvik, 2012). There is a need for further research into the possibilities of enhancing insight, acceptance, and attunement to new possibilities among people with troublesome voice hearing through working with narratives, oral or written.

Our results reveal that most participants developed new ways of approaching the voices and sounds and that they managed to grasp possibilities and resources. Nonetheless, many participants recurrently turned to previously unhelpful or hurtful strategies when intensely troubled. These results corroborate Romme and Escher's (1989) suggestion that patients developed “more continuous way of dealing with voices” and Sayer, Ritter, and Gournay's (2000) indication that neither coping nor attribution styles become more stable over time. We suggest with Benner and Wrubel (1989) that people struggling with health issues such as being troubled by voices and sounds “enter into situations, with their own sets of meanings, habits, and perspectives. And the particular ways of being in the situation set up particular lines of action and possibilities” (p. 23).

The validity of the results rests on the quality of the research process and the findings (Whittemore, Chase, & Mandle, 2001). We established that the quality of the interviews and the number of participants were appropriate to answer the research question. The interviewees had broad, varied, and relevant experiences and reflections, which they were eager to convey. The analysis was concluded when the interpretations of the entirety of the interview texts and the parts of the texts corresponded (Fleming et al., 2003) and we had developed a thorough and mutual understanding over a substantial time period (Gonzalez, 2006). At subsequent presentations and in dialogs about the findings, other voice hearers, relatives, and health carers have confirmed that the results were relevant. They also challenged the authors’ presumptions and thus contributed to the continuing exclusion of erroneous understanding and provided new sources of understanding (cf. Gadamer, 2004, p. 298). We therefore believe that the results of this study can be relied upon to enrich our understanding of how people deal with hearing voices and sounds in everyday life.

## Concluding reflections and implications

Our study supports the need for exploring how people with mental illness deal with hearing voices and sounds under shifting phases and circumstances in daily life. The participants’ strategies were not restricted to managing symptoms of a mental illness. The participants struggled to manage everyday life because of, and despite, hearing voices. They clung to meaningful activities to fill their lives with something other than the voices and sounds. They struggled to remain independent despite recurrently being dependent on health care services and attempted to diminish the burden of the undesirable effects of medication. We suggest that attempting to understand how people with mental illness deal with hearing voices should be fundamental to health care for this patient group. Health care providers should carefully assess how the voice hearer has managed everyday life in different phases and under shifting circumstances. The voice hearer's struggle should be recognized and acknowledged. Person-centered care and shared-decision making are approaches that address important aspects of empowering relationships between care providers and service users. Health care providers should realize the importance of assisting voice hearers in the process of coming to terms with and making sense of hearing voices along with other adversities in their lives and also be aware of the possibilities offered by peer support groups. Further research should address how people deal with voices over time and in different phases, the goals they have in dealing with them, and how they evaluate and understand these strategies.
